# Strength–Endurance Training Reduces Tremor Severity and Improves Manual Dexterity and Upper Extremity Function in Adults with Essential Tremor: A Quasi-Experimental Study

**DOI:** 10.3390/life16060961

**Published:** 2026-06-06

**Authors:** Cemal Polat, Ali Muhittin Taşdoğan, Emre Yavuz, Zarife Pancar

**Affiliations:** 1 Department of Coaching Education, Faculty of Sports Sciences, Eskişehir Technical University, 26555 Eskişehir, Turkey; cpolat@eskisehir.edu.tr; 2 Department of Anesthesiology and Reanimation, Faculty of Medicine, Gaziantep University, 27310 Gaziantep, Turkey; drmtasdogan@gmail.com; 3 Department of Physical Education and Sports, Institute of Health Sciences, Gaziantep University, 27310 Gaziantep, Turkey; emreyavuz1023@gmail.com; 4 Department of Physical Education and Sports, Faculty of Sports Science, Gaziantep University, 27310 Gaziantep, Turkey

**Keywords:** essential tremor, strength–endurance, manual dexterity, exercise intervention, upper extremity function

## Abstract

Essential tremor (ET) is among the most prevalent movement disorders, causing significant impairment in manual dexterity and daily functioning. Although ET affects individuals across the lifespan, exercise intervention research has focused almost exclusively on older adults, leaving young adults, for whom early intervention may prevent long-term neuromuscular deterioration, critically underrepresented. Furthermore, the effects of strength–endurance oriented exercise combined with task-specific motor activities remain insufficiently explored in any ET population. This quasi-experimental pre-test–post-test study investigated the effects of a 6-week progressive strength–endurance and task-specific exercise program on tremor severity, manual dexterity, and upper extremity functional performance in young adult males with ET (n = 18; mean age: 22.6 ± 4.1 years). The 24-session intervention (four sessions/week) combined proximal upper extremity strength–endurance exercises with seven ADL-specific fine motor tasks. Tremor severity was assessed using the Fahn–Tolosa–Marin Tremor Rating Scale (FTMTRS), manual dexterity using the Nine-Hole Peg Test (NHPT), and upper extremity stability using the Closed Kinetic Chain Upper Extremity Stability Test (CKCUEST). The Wilcoxon signed-rank test was used for within-group comparisons, with rank biserial correlation (r) and Cohen’s d reported as effect size indices. Significant pre-to-post improvements were observed across nearly all outcome measures, with medium-to-large effect sizes. Spiral drawing performance improved significantly in five of six conditions (r = 0.47–0.62), with the exception of the Spiral left–B task (*p* = 0.083). Postural tremor, NHPT (both hands), and CKCUEST also showed significant improvements (r = 0.47–0.73). A composite tremor score, integrating all tremor sub-scores, demonstrated a 14.1% overall reduction (*p* = 0.001, r = 0.83), providing strong evidence of program-wide effectiveness. Session adherence was 95.8%. To our knowledge, this is one of the first studies to show that a structured strength–endurance and task-specific exercise program was associated with reductions in tremor severity and improvements in upper extremity function, specifically in young adults with ET. These findings support the clinical utility of exercise as a non-pharmacological intervention in this underserved population and highlight the importance of early, targeted intervention during young adulthood.

## 1. Introduction

Tremor refers to an involuntary, repetitive, rhythmic movement affecting one or more body segments [1]. Under normal conditions, unsupported limbs and the head may exhibit a mild physiological tremor, which is typically not visible or clinically significant unless exacerbated by factors such as fatigue or anxiety [2]. From a topographical perspective, tremor most frequently affects the hands (94%), followed by the head (33%), voice (16%), jaw (8%), face (3%), legs (12%), and trunk (3%) [3]. Essential tremor (ET) is characterized by bilateral action tremor predominantly affecting the upper limbs and ranks among the most common movement disorders encountered in clinical practice [1,4]. Epidemiological data indicate that ET affects approximately 1% of the general population and up to 4–5% of individuals over the age of 65 [3]. Importantly, however, ET is not exclusively a disease of older age groups. Among adults aged 18 and over residing in Turkey, population-based surveys have estimated a prevalence of 3.09% [5], and accumulating evidence suggests that a non-negligible proportion of younger adults, including university students and young working-age individuals, may present with clinically relevant ET symptoms that significantly impair daily functioning [6]. Despite this, the vast majority of exercise intervention studies targeting ET have focused exclusively on older adult populations, leaving the young adult age group substantially underrepresented in the literature. ET is a progressive condition; if left unmanaged during the earlier stages of life, neuromuscular maladaptations, including increased variability in motor unit discharge rates, excessive co-activation between agonist and antagonist muscles, and disrupted motor unit synchronization, may become further entrenched over time [7,8]. Early intervention during young adulthood may therefore represent a critical window for attenuating disease progression and preserving functional capacity before these adaptations become irreversible. Furthermore, fatigue has been identified as a critical factor influencing tremor dynamics. Studies in healthy young individuals have demonstrated that fatigue can significantly increase tremor amplitude, particularly within the 8–12 Hz frequency band, ultimately impairing task performance [9,10,11]. Localized fatigue in a single limb segment has been shown to affect tremor not only in adjacent segments but also in contralateral limbs, suggesting the presence of complex neuromuscular and central regulatory mechanisms [9,12].

Both resting and postural tremors significantly interfere with activities of daily living (ADL), often leading to functional limitations and psychological distress, particularly in social contexts where symptoms become more visible [13,14]. Tasks most affected by tremor include handwriting, eating, dressing, and personal hygiene, all of which require fine motor skills involving precise and coordinated movements of small muscle groups in the hands and fingers [15]. In young adults, these limitations carry a dimension of impact that extends beyond physical disability. Tremor-related impairments frequently interfere with academic performance, including note taking, laboratory work, and computer use, as well as with professional skill development and social participation during a formative period of life. Psychological consequences, including anxiety in academic or social set-tings and reduced self-efficacy, may further compound the functional burden [16]. Despite the considerable impact of ET on quality of life in this age group, targeted non-pharmacological interventions developed specifically for young adults with ET remain conspicuously absent from the literature. Fine motor skills are essential for executing complex, goal directed actions that rely on accuracy, dexterity, and neuromuscular coordination [13]. Addressing these deficits through structured exercise interventions in early adulthood could meaningfully reduce long-term functional decline and improve quality of life in this underserved population.

In recent years, growing evidence has highlighted the potential role of physical exercise as a non-pharmacological strategy for managing tremor-related impairments. Regular physical activity has been shown to improve both physiological and psychological parameters associated with tremor [17]. Within this context, resistance training has emerged as one of the most commonly applied exercise modalities in tremor management programs [18]. Previous studies involving individuals with ET have reported significant improvements in manual dexterity following exercise based interventions, as evidenced by enhanced performance in the Purdue Pegboard Test, Nine-Hole Peg Test, Moberg Pick-Up Test, and Jebsen Hand Function Test [19]. These improvements have been observed in both the more-affected and less-affected limbs, suggesting that exercise interventions may induce widespread neuromuscular adaptations contributing to improved motor control. Notably, previous studies have demonstrated that high load resistance training programs targeting upper extremity muscle groups over a 6-week period can lead to meaningful reductions in tremor amplitude and improvements in motor performance [20]. Neural mechanisms underlying these adaptations, including enhanced motor unit recruitment, reduced discharge rate variability, and improved sensorimotor integration, are not age-dependent phenomena; they have been demonstrated in both young and older adults following structured exercise exposure [21,22]. This supports the rationale that exercise-induced neuromuscular adaptations are equally plausible in young adults with ET. However, a critical limitation of the existing literature is that exercise protocols have predominantly targeted older individuals (aged 60 years and above), and have focused primarily on strength development rather than fatigue resistance or sustained motor control. Systematic review evidence further suggests that most protocols for ET involve 6-week interventions twice per week using dumbbell-based resistance exercises at moderate intensity (40–60% of maximum capacity) [23]. Despite the demonstrated benefits, the effects of strength–endurance-oriented and task-specific exercise, designed to build fatigue resistance, improve interlimb coordination, and sustain motor control under load, remain insufficiently explored, particularly in young adults for whom such functional capacities are critical for daily academic and occupational performance.

It is therefore hypothesized that upper extremity-focused strength–endurance exercises, combined with task-specific motor activities targeting ADL-relevant skills, may enhance fatigue resistance, reduce interlimb asymmetry, improve manual dexterity, and ultimately decrease tremor severity in young adults with essential tremor. The present study addresses a clear gap in the literature by being, to our knowledge, one of the first to examine the effects of a structured strength–endurance and task-specific exercise program specifically in a young adult ET population. Accordingly, the aim of this study was to investigate the effects of a 6-week progressive strength–endurance and daily physical activity-based exercise program on tremor severity, manual dexterity, and upper extremity functional performance in young adults with essential tremor.

## 2. Materials and Methods

### 2.1. Study Design

This study employed a single-group quasi-experimental pre-test–post-test design. A randomized controlled trial design was considered; however, given the limited availability of young adults with confirmed ET diagnoses and the ethical considerations associated with withholding a potentially beneficial intervention from this population, a single-group design was deemed the most feasible and appropriate approach for this initial investigation [24,25]. Participants with essential hand tremor aged 17–35 years were recruited using purposive sampling. Based on comparable pilot studies in the literature, which typically report sample sizes ranging from 10 to 30 participants with a median of approximately 18 [24,25], a target sample of 20 was considered appropriate for detecting meaningful within-subject changes while maintaining feasibility. A total of 20 participants were initially enrolled; 18 completed the full intervention and were included in the final analysis. Assessments were conducted at two time points: baseline (pre-intervention) and immediately following the six-week intervention (post-intervention).

### 2.2. Participants

**Figure 1:**
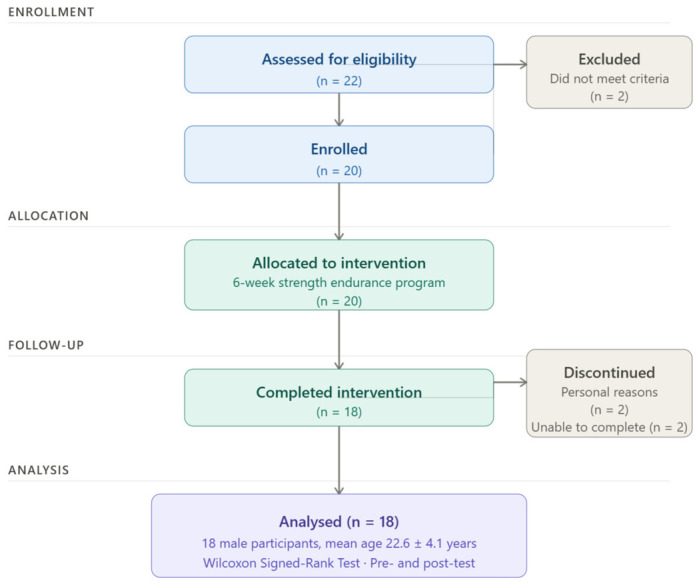
Participant flow diagram.

Participants were recruited using purposive sampling. Inclusion criteria were: (1) aged 17–35 years; (2) diagnosis of essential tremor confirmed by a neurologist; (3) prominent upper extremity tremor (particularly in the hand and wrist); (4) self-reported functional limitations in daily living activities due to tremor; (5) no participation in a regular upper extremity exercise program in the preceding year; (6) sufficient cognitive capacity to follow the exercise program independently; and (7) no recent history of shoulder, elbow, or wrist injury. Exclusion criteria included: (1) presence of other neurological conditions such as Parkinson’s disease; (2) history of orthopedic injury or surgery affecting the upper extremity; (3) severe musculoskeletal pain or restricted range of motion; (4) severe visual or balance impairment; (5) advanced cognitive impairment; or (6) any change in tremor-related medication dosage during the study period. Of the 20 participants initially enrolled, two withdrew for personal reasons following pre-testing and two were unable to complete the intervention. The study was completed with 18 male participants (mean age: 22.6 ± 4.1 years) (Figure 1). Descriptive statistics of the participants are shown in Table 1. The inclusion of only male participants was a deliberate methodological decision to ensure sample homogeneity and to minimize the potential confounding influence of hormonal variation on neuromuscular function and tremor characteristics, which has been reported to differ between sexes [6]. All participants reported right-hand dominance. All participants provided written informed consent prior to enrollment and were informed of their right to withdraw at any time. Pre- and post-tests were conducted at the Biomechanics Research Laboratory of Eskişehir Technical University. Participants were instructed to refrain from alcohol, nicotine, and caffeine on test days. Ethical approval for this study was obtained from the Scientific Research and Publication Ethics Committee of Eskişehir Technical University, Faculty of Science and Engineering (Approval No: 62641; Date: 15 January 2025). The study was conducted in accordance with the Declaration of Helsinki. Prior to study entry, all participants gave written informed consent and were made aware that participation was voluntary and could be discontinued at any time.

### 2.3. Exercise Protocol

Participants completed a six-week progressive strength–endurance and task-specific therapeutic exercise program, consisting of four sessions per week, each lasting approximately 45–60 min. The program was structured in two progressive loading blocks and designed to target fine motor control, manual dexterity, and upper extremity muscle endurance. The first block (Sessions 1–12, Weeks 1–3) included a 10 min warm-up (jumping jack variations), followed by two main phases: (1) a 20 min fine and gross motor skill component comprising seven task-specific activities: ball squeezing, finger tapping, block stacking, button fastening/unfastening, handwriting, cutlery use, and water pouring/emptying, performed for 7 × 30 s × 4 sets with 6 min total rest; and (2) a 20 min strength–endurance component involving five upper extremity exercises (Side Wall Plank, Side Plank with Circle-Out and Knee Curl, Floating Diamond Plié Squat with Forward Push, Torso Rotation with Knee-Up, and Reverse Plank), performed for 2 sets of 10 repetitions or 30 s with 1 min inter-set rest. Each session concluded with a 10 min active cool-down (flexibility exercises). The session Rating of Perceived Exertion (sRPE) for this block was targeted at 4–5 au on the Borg–Foster scale. The second block (Sessions 13–24, Weeks 4–6) followed the same structure but with increased training volume (additional sets and repetitions) to achieve progressive overload, targeting an sRPE of 6–7 au. The first two sessions were conducted under the supervision of a graduate-level exercise specialist. Remaining sessions were performed independently at home or on campus. Weekly adherence monitoring was conducted via telephone or written communication, through which participants self-reported completed sessions using a structured session log provided at the start of the intervention. Adherence was defined as completion of the prescribed exercises for the full duration of each session. No objective monitoring device was used to verify exercise execution quality; thus, adherence data relied primarily on participant self-report, which represents a potential limitation of the home-based delivery format. Participant compliance was tracked throughout the intervention period. Overall session adherence across all participants was 95.8% (mean sessions completed: 23.0 ± 0.9 out of 24), indicating a high level of compliance with the prescribed exercise program.

The five strength–endurance exercises were selected to target the proximal upper extremity musculature—specifically the shoulder girdle, scapular stabilizers, and core—based on evidence that proximal stability is a key determinant of distal motor control and tremor attenuation. The seven task-specific fine motor activities were chosen to directly correspond to the daily living tasks most frequently impaired in individuals with essential tremor, including handwriting, eating, and personal hygiene [13,15]. Exercise selection was further guided by the principle that repetitive, controlled motor tasks may enhance sensorimotor integration and reduce motor unit discharge rate variability through neuroplastic adaptation [21,22].

#### Outcome Measures

All assessments were conducted under standardized laboratory conditions at the Faculty of Sports Sciences, Eskişehir Technical University. Measurements were per-formed at baseline (pre-test, week 1) and after completion of the intervention (post-test, week 6).

### 2.4. Tremor Severity—Fahn–Tolosa–Marin Tremor Rating Scale (FTMTRS)

Tremor severity was quantified using the FTMTRS, a clinically validated rating tool that evaluates tremor amplitude across body regions using a five-point ordinal scale ranging from 0 (no tremor) to 4 (severe tremor) [26]. In this study, the postural hand tremor component and the Archimedes spiral drawing task (FTMTRS Section B; Drawings A, B, and C) were used as outcome measures. Postural tremor severity was assessed using a standardized video recording protocol. Participants sat with hips and knees flexed at 90°, arms fully extended forward, wrists slightly extended, and fingers comfortably abducted. Tremor amplitude and severity were independently rated by a trained neurologist using a 5-point scale: 0 = normal; 1 = mild tremor (amplitude < 0.5 cm); 2 = moderate tremor (0.5–1 cm); 3 = marked tremor (1–2 cm); 4 = severe tremor (>2 cm) [26]. For the Archimedes Spiral Test, participants were instructed to connect marked points to complete spiral shapes without crossing the boundary lines. Each hand was assessed separately, beginning with the less-affected side. Participants were instructed not to rest their hands or arms on the table to prevent external stabilization. Drawings were scored using a standardized 5-point system: 0 = normal; 1 = mild tremor occasionally crossing boundary lines; 2 = frequent boundary crossings; 3 = marked difficulty with multiple errors but task completed; 4 = unable to complete due to severe tremor [26]. Video recordings from both pre- and post-test assessments were presented to the neurologist in a randomized order, without explicit labeling of testing timepoint. However, formal blinding to intervention status was not implemented, as the assessor was aware that participants had undergone an exercise intervention. The FTMTRS demonstrates good inter-rater reliability (interclass correlation *p* = 0.97) and excellent intra-rater reliability across repeated assessments [27].

#### 2.4.1. Manual Dexterity: Nine-Hole Peg Test (NHPT)

Fine motor performance was evaluated with the NHPT, a timed pegboard task widely adopted in clinical and research settings as a reliable index of hand dexterity. The test apparatus consisted of a nine-hole pegboard and nine cylindrical pegs (diameter: 0.64 cm). The NHPT demonstrates high inter-rater reliability (right hand r = 0.97; left hand r = 0.99) and acceptable test–retest reliability [28]. Participants were seated with the pegboard positioned at midline and the peg container placed on the side of the hand being tested. The dominant hand was assessed first. Following a brief demonstration, participants were instructed to insert and remove all nine pegs as quickly as possible using the designated hand. Timing began when the participant touched the first peg and ended when the last peg was returned to the container. Total completion time (in seconds) was recorded for each hand [28,29]. For reference, normative NHPT completion times for healthy adults aged 20–24 years have been reported as approximately 18.0 s for the dominant hand and 19.9 s for the non-dominant hand [29], providing a contextual benchmark against which participants’ baseline performance was interpreted.

#### 2.4.2. Upper Extremity Stability: Closed Kinetic Chain Upper Extremity Stability Test (CKCUEST)

**Figure 2:**
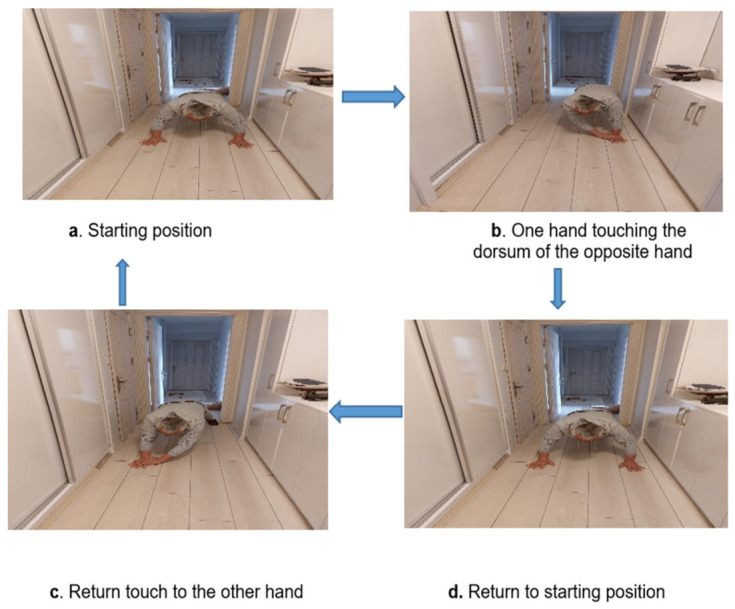
Closed Kinetic Chain Upper Extremity Stability Test (CKCUEST) sequence. (**a**) Starting position: participant assumes a modified push-up position with knee support on a flat, non-slip surface, hands placed slightly wider than shoulder width and 91.4 cm apart. (**b**) Right hand crosses the midline to touch the dorsum of the left hand. (**c**) Left hand crosses the midline to touch the dorsum of the right hand. (**d**) Return to starting position. The green marker indicates the touch point. One complete repetition consists of steps from (**b**,**c**). Two 15 s trials were performed with a 60 s rest interval between trials (work-to-rest ratio 1:4).

Upper extremity functional performance, including strength, stability, and muscular endurance, was evaluated using the CKCUEST, a valid and reliable field-based assessment (ICC = 0.92–0.97) [30,31,32]. Participants assumed a modified push-up position on a flat, non-slip surface with knee support, hands placed slightly wider than shoulder width and approximately 91.4 cm apart (Figure 2). Each repetition involved extending one hand across the body midline to make contact with the back of the opposite hand and then returning to the initial position. Participants completed as many repetitions as possible within a 15 s period. Each participant performed two attempts separated by a 60 s recovery period, yielding a work-to-rest ratio of 1:4, and the highest score was retained for analysis. To account for body mass differences, a relative performance score was calculated as follows: (total touches × 0.68)/body mass (kg). Normative values in healthy young adults range from approximately 22 to 31 repetitions per 15 s, with higher values reported in athletic populations [33,34].

#### 2.4.3. Session Rating of Perceived Exertion (sRPE)

Internal training load was quantified via the sRPE method, originally formulated by Borg [35] and subsequently adapted for exercise training contexts by Foster [36]. At 30 min post-session, each participant provided a global exertion rating on a 0–10 scale in response to a standardized prompt regarding overall workout intensity. Given the two-phase progressive loading design, sRPE values were calculated separately for the end of Session 1, Session 12, and Session 24.

### 2.5. Statistical Analysis

All statistical analyses were performed using SPSS version 25.0 (IBM Corp., Ar-monk, NY, USA). Descriptive statistics were reported as median, mean rank, and 95% bias-corrected and accelerated bootstrap confidence intervals (BCa CI; 1000 iterations). Normality of the distribution was examined using the Shapiro–Wilk test applied to pre-to-post difference scores for each variable. As the assumption of normality was violated for the majority of variables, within-group changes from pre- to post-intervention were examined using the Wilcoxon signed-rank test. In addition to statistical significance, effect size was calculated for each comparison using the rank biserial correlation coefficient (r = Z/√N), with thresholds of 0.10, 0.30, and 0.50 denoting small, medium, and large effect magnitudes, respectively [37,38]. Statistical significance was defined as *p* < 0.05 throughout. To complement the nonparametric effect size estimates, Cohen’s d was additionally calculated for each comparison as a standardized parametric effect size index, using the pooled standard deviation of pre- and post-test scores. Values of 0.20, 0.50, and 0.80 were interpreted as small, medium, and large effects, respectively [37].

## 3. Results

**Figure 3:**
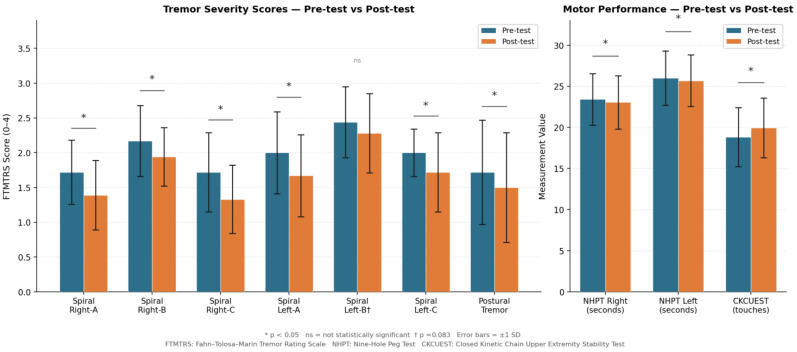
Pre- and post-test mean scores (±1 SD) for tremor severity and motor performance outcomes. Left panel: Fahn–Tolosa–Marin Tremor Rating Scale (FTMTRS) scores for spiral drawing tasks and postural hand tremor. Right panel: Nine-Hole Peg Test (NHPT) completion time (seconds) and Closed Kinetic Chain Upper Extremity Stability Test (CKCUEST) touch count. Statistically significant pre-to-post differences are indicated by asterisks (* *p* < 0.05). The Spiral left–B condition did not reach statistical significance (^†^ *p* = 0.083). Error bars represent ±1 SD.

The Wilcoxon signed-rank test results indicated statistically significant pre-to-post-test differences in postural hand tremor, Spiral right −A, −B, −C; Spiral left −A, −C; NHPT right hand; NHPT left hand; CKCUEST; respectively (Z = −2.000, *p* = 0.046, r = 0.47; Z = −2.449, *p* = 0.014, r = 0.57; Z = −2.000, *p* = 0.046, r = 0.47; Z = −2.646, *p* = 0.008, r = 0.62; Z = −2.449, *p* = 0.014, r = 0.57; Z = −2.236, *p* = 0.025, r = 0.52; Z = −2.276, *p* = 0.023, r = 0.53; Z = −2.246, *p* = 0.025, r = 0.52; Z = −3.109, *p* = 0.002, r = 0.73). A high effect size was observed in CKCUEST variables (r > 0.70). No statistically significant difference was found in the Spiral left–B variable (Z = −1.732, *p* = 0.083, r = 0.40). The session sRPE values at the end of Session 1, Session 12, and Session 24 were 4.2, 4.9, and 5.3 (au), respectively, reflecting a progressive increase in training load across the intervention (Table 2, Figure 3). These findings suggest that the intervention produced meaningful effects across nearly all assessed variables.

**Table 2:** Within-group pre- and post-test comparisons of tremor severity, hand skill, and muscle strength variables (Wilcoxon signed-rank test).

Variables	Z	*p*	r	Cohen d
Postural hand tremor	−2.0	0.046 *	0.47	−0.29
Spiral right–A	−2.449	0.014 *	0.57	−0.69
Spiral right–B	−2.0	0.046 *	0.47	−0.48
Spiral right–C	−2.646	0.008 **	0.62	−0.73
Spiral left–A	−2.449	0.014 *	0.57	−0.56
Spiral left–B	−1.732	0.083	0.40	−0.31
Spiral left–C	−2.236	0.025 *	0.52	−0.59
NHPT-right hand	−2.276	0.023 *	0.53	−0.11
NHPT-left hand	−2.246	0.025 **	0.52	−0.10
CKCUEST	−3.109	0.002 **	0.73	+0.31
Composite Tremor Score	−3.516	0.0004 **	0.83	−0.68

*p* < 0.05 *, *p* < 0.01 ** NHPT: Nine-Hole Peg Test; CKCUEST: Closed Kinetic Chain Upper Extremity Stability Test.

## 4. Discussion

The present investigation evaluated the impact of a six-week progressive training program combining strength–endurance and task-specific exercise on tremor severity, fine motor performance, and upper limb functional capacity in young adults diagnosed with ET, a population that has been largely overlooked in the existing exercise intervention literature. It was hypothesized that increasing muscular endurance capacity in the shoulder, arm, and hand, combined with task-specific motor training, would attenuate tremor amplitude, enhance sensorimotor integration, and improve fine motor performance. The findings broadly support this hypothesis, demonstrating statistically significant pre-to-post improvements across nearly all outcome measures (*p* < 0.05 to *p* < 0.002), with medium-to-large effect sizes (r = 0.47–0.73, d = −0.11 to −0.73). This represents one of the first investigations to document the effects of a strength–endurance and task-specific exercise program specifically within a young adult ET population, thereby extending the current evidence base beyond its predominant focus on older adults.

Significant improvements were observed in postural hand tremor and Archimedes spiral performance (right hand A–C; left hand A and C), consistent with previous studies reporting that resistance and task-specific exercise effectively reduces tremor amplitude and severity [20,21,22,23,39,40,41]. Previous studies suggest that resistance exercise may contribute to improved motor control and tremor reduction through adaptations in neuromuscular coordination and force regulation [20,21,22,23]. However, these mechanisms were not directly assessed in the present study and should therefore be interpreted cautiously.

It should also be acknowledged that repeated exposure to spiral drawing and dexterity testing may itself have contributed to performance improvements independent of the exercise intervention, as test familiarity and motor learning effects cannot be excluded in the absence of a control group. The absence of significant change in the Spiral left–B task (r = 0.40, *p* = 0.083) may reflect the higher visuomotor integration and hand eye coordination demands of this specific drawing condition, which may be more sensitive to exercise volume, intensity, or intervention duration. This observation suggests that more complex drawing tasks may require longer training periods or coordination-focused interventions to yield measurable improvements.

Although the mechanisms responsible for the observed improvements cannot be determined from the present data, the previous literature suggests that structured exercise may support motor control and functional performance in individuals with ET [4,7,21,42,43]. Given the relatively young age of the participants, the observed improvements may reflect favorable neuromuscular adaptations; however, direct neurophysiological measurements were not performed and therefore mechanistic interpretations remain speculative.

Significant improvements in NHPT performance (right and left hand) suggest improvements in fine motor precision and sensorimotor coordination following the intervention. The NHPT requires continuous proprioceptive feedback and motor adaptation, both of which may be augmented by the repetitive, structured nature of resistance exercise [22,23]. These gains are consistent with findings from studies in individuals with essential tremor, in which structured resistance training yielded consistent gains in hand dexterity and tremor control [44,45]. From a functional standpoint, such gains may support performance of everyday tasks including meal preparation, written communication, and self-care activities, which are among the most commonly affected tasks in individuals with essential tremor [13,14]. It should be acknowledged, however, that the absolute magnitude of NHPT improvement in the present study was modest (right hand: −0.36 s; left hand: −0.31 s), representing percentage improvements of 1.5% and 1.2% for the right and left hands, respectively, and the corresponding Cohen d values were small (d = −0.11 and −0.10, respectively). Although these differences reached statistical significance, they fall below the commonly reported minimally important difference (MID) threshold of approximately 2.0 s for the NHPT in neurological populations. This discrepancy between statistical and clinical significance highlights the importance of interpreting NHPT findings cautiously in this population, and is likely attributable to the relatively preserved baseline dexterity of young participants, whose pre-test NHPT times (right: 23.4 s; left: 26.0 s) were only modestly elevated compared to age-matched normative values (approximately 18.0 s and 19.9 s, respectively), leaving limited room for improvement within the six-week timeframe. Future studies should consider longer intervention durations or more sensitive fine motor outcome measures to better capture clinically meaningful changes in this population.

Notably, among all outcome measures, the CKCUEST demonstrated the largest and most clinically persuasive effect size (r = 0.73), and should therefore be considered the primary indicator of intervention-induced functional improvement in the present study. This finding aligns with the progressive overload principle applied across the two training blocks, whereby systematic increases in training volume (sets and repetitions) may have facilitated neuromuscular adaptations, including improved intramuscular and intermuscular coordination. Resistance exercises incorporating fine motor components may also promote improvements in hand dexterity and finger range of motion [46]. Improvements in CKCUEST performance may indicate enhanced upper extremity stability and functional capacity. It is possible that improved proximal control contributed to better distal motor performance; however, this relationship was not directly examined in the present study and should be interpreted with caution [47]. It should be noted, however, that the present study did not directly measure proximal stability or its relationship to distal tremor expression, and these mechanistic interpretations therefore remain speculative. These proposed mechanisms, while not directly tested in the present study, suggest that exercises targeting proximal strength–endurance may constitute an important contributor to functional tremor reduction, particularly in young adults for whom the neuromuscular system retains high adaptability.

To further contextualize these findings, a responder analysis was conducted to determine the proportion of participants demonstrating individual improvement across key outcome measures. Results indicated that 14 of 18 participants (77.8%) showed improvement in NHPT left hand, 13 of 18 (72.2%) in CKCUEST, and 12 of 18 (66.7%) in NHPT right hand. In contrast, only 4 of 18 participants (22.2%) demonstrated improvement in postural tremor, a finding consistent with the moderate effect size observed for this variable (r = 0.47, d = −0.29). This discrepancy is likely attributable to a ceiling effect in the postural tremor rating scale, where many participants were already scored at mild severity (1–2 on the FTMTRS 0–4 scale) at baseline, limiting the detectable range of improvement. These responder rates align with the overall pattern of statistically significant pre-to-post changes and support the clinical relevance of the intervention. A composite tremor score, calculated as the mean of all spiral drawing and postural tremor sub-scores, further demonstrated a significant overall reduction following the intervention (1.97 ± 0.29 vs. 1.69 ± 0.25; Z = −3.516, *p* < 0.001, r = 0.83, d = −0.68), suggesting program-wide improvements across the full spectrum of tremor-related outcomes assessed. Progressive increases in sRPE values across the intervention (Session 1: 4.2 au; Session 12: 4.9 au; Session 24: 5.3 au) suggest that the two-phase loading design was successfully implemented and perceived as progressively more demanding by participants. This is consistent with the session-RPE method as a valid indicator of internal training load in structured exercise programs [36].

A number of methodological constraints must be considered when drawing conclusions from these findings. First, the absence of a control group precludes definitive causal inference; observed improvements could reflect practice effects, natural fluctuation in tremor severity, or regression to the mean rather than a true intervention effect. Second, the relatively small sample size (n = 18) reduces statistical power and increases the risk of both Type II error and inflated effect size estimates. Third, the single-sex composition (all male) restricts the generalizability of findings to female individuals with ET. Sex differences in tremor presentation, neuromuscular adaptation, and response to exercise interventions are well documented in the literature. For instance, females may exhibit different patterns of motor unit recruitment, lower absolute muscle endurance capacity, and distinct hormonal influences on neuromuscular function that could meaningfully alter the magnitude and direction of training-induced adaptations. Furthermore, ET prevalence and severity have been reported to differ between sexes, with some evidence suggesting that females may present with more severe postural tremor. These factors collectively suggest that the findings of the present study cannot be assumed to generalize to female individuals with ET, and future studies should prioritize the inclusion of female participants to establish sex-specific response patterns to strength–endurance training. Fourth, the predominantly home-based delivery of sessions (22 of 24) limits the degree of exercise quality control; despite weekly monitoring, the accuracy of exercise execution could not be objectively verified. Fifth, tremor severity was assessed using a clinical ordinal rating scale (FTMTRS), which, while widely used, lacks the sensitivity and objectivity of accelerometers or electromyography-based quantification. The ceiling and floor effects inherent to ordinal scales may have obscured true changes in tremor amplitude, particularly in participants with mild baseline severity. Sixth, tremor severity ratings were performed by a trained neurologist; however, the assessor was not formally blinded to testing timepoint or intervention status. Although video recordings were presented in randomized order, formal blinding to intervention status was not implemented, which introduces a potential risk of expectation bias in the primary outcome assessment. This represents an important methodological limitation that should be addressed in future studies through blinded and randomized scoring procedures. Seventh, the absence of a follow-up assessment prevents conclusions regarding the durability of intervention effects beyond the immediate post-test period. Future studies should incorporate randomized controlled designs with larger, more diverse samples including female participants, longer intervention and follow-up periods, and objective tremor quantification using wearable sensor technology. The potential benefits of combining strength–endurance training with coordination-focused or visuomotor interventions in young adult ET populations also warrant further systematic investigation.

## 5. Conclusions

A six-week progressive strength–endurance and task-specific exercise program was associated with improvements in tremor severity, manual dexterity, and upper extremity functional performance in young adults with essential tremor. High participant adherence and the feasibility of partial home-based delivery suggest that this approach may represent a practical complementary strategy for this population. However, given the quasi-experimental design, the absence of a control group, and the relatively small sample size, these findings should be considered preliminary. Larger randomized controlled trials with objective tremor assessment methods and longer follow-up periods are required to determine the effectiveness and long-term applicability of this intervention.

## Figures and Tables

**Table 1:** Baseline demographic and anthropometric characteristics of the participants.

Variable		95% Confidence Interval	
	M ± SD	Lower	Upper
**Age (years)**	22.61 ± 4.13	20.83	24.55
**Height (cm)**	172.1 ± 4.58	167.1	172.2
**BW (kg)**	70.7 ± 8.03	67.1	74.4
**BMI (kg/m^2^)**	23.89 ± 2.42	19.89	27.47

BMI: Body mass index.

## Data Availability

The data are presented within the article and that raw data are available upon reasonable request due to ethical and privacy restrictions involving human participants.
